# The first report of *Staphylococcus argenteus* endocarditis with visualized valve vegetations

**DOI:** 10.1016/j.idcr.2024.e02058

**Published:** 2024-08-14

**Authors:** Ebba Hillstedt, Magnus Dencker, Bo Nilson, Karl Oldberg, Magnus Rasmussen

**Affiliations:** a Department of Perioperative Medicine and Intensive Care Medicine, Skåne University Hospital, Malmö, Sweden; b Department of Infectious Diseases, Skåne University Hospital, Malmö, Sweden; c Department of Medicine, Nyköping Hospital, Sörmland Region, Nyköping, Sweden; d Centre for Clinical Research Sörmland, Uppsala University, Sweden; e Department of Medical Imaging and Physiology, Skåne University Hospital, Sweden; f Department of Translational Medicine, Lund University, Malmö, Sweden; g Department of Clinical Microbiology, Infection Prevention and Control, Office for Medical Services, Region Skåne, Sweden; h Division of Medical Microbiology, Department of Laboratory Medicine, Lund University, Lund, Sweden; i Department of Clinical Sciences Lund, Division of Infection Medicine, Lund University, Lund, Sweden; j Department of Infectious Diseases, Skåne University Hospital, Lund, Sweden

**Keywords:** Infective endocarditis, Endocarditis, *Staphylococcus argenteus*, Sepsis, Echocardiography, Bacteremia

## Abstract

*Staphylococcus argenteus* was recently defined as a species and has previously often been mistaken for *Staphylococcus aureus* due to the difficulties of conventional laboratory methods to distinguish the two species. The clinical presentation of infections caused by *S. argenteus* is largely unknown, and its virulence has since the definition of the species been debated. Here we present, to our knowledge, the first case of infective endocarditis due to *S. argenteus* with valve vegetations visualized on echocardiography. The 74-year-old male patient with biological aortic valve prothesis presented with a rapid onset of diffuse symptoms and his condition hastily deteriorated to septic shock followed by several complications such as intracranial septic emboli, severe heart failure, and intracardiac thrombus. After conservative treatment and management of the multiple complications, the patient recovered and was eventually discharged to his original housing situation.

## Introduction

*Staphylococcus argenteus* is a coagulase-positive *Staphylococcus* species formally defined as late as in 2015 [1]. The species was initially described as a clonal complex (*Staphylococcus aureus* clonal complex 75) of the more widely known *Staphylococcus aureus* when first described in 2009. It has later been redefined as a part of the *S. aureus*-related complex together with *S. aureus* and *Staphylococcus schweitzeri* [1], [2]. The differentiation between *S. argenteus* and *S. aureus* is challenging, implying that *S. argenteus* often has been misclassified as *S. aureus.* The two bacterial species have near identical 16S rRNA sequences and conventional biochemical analyses fail to differentiate between *S. argenteus* and *S. aureus* [3]. Matrix-assisted laser desorption ionization-time of flight mass spectrometry (MALDI-TOF MS) can reliably identify *S. argenteus/S. schweitzeri* group of species from *S. aureus* if they are represented with mass spectra in the reference database. It is more difficult to differentiate *S. argenteus* and *S. schweitzeri* from each other but species-specific mass peaks have been identified that can be used to separate them [1], [2].

The pathogenicity of *S. argenteus* has been debated since its first isolation, with initial reports hypothesizing that *S. argenteus* has a lower virulence than *S. aureus* [4]*.* Novel publications implicate that *S. argenteus* potentially is as virulent as *S. aureus,* with one study even demonstrating a higher mortality risk for *S. argenteus* than for *S. aureus* infections [2], [5].

According to our literature search there is only one prior published case of infective endocarditis (IE) due to *S. argenteus*, in which the diagnosis was based on microbiological major criterium and three minor criteria. The echocardiography, however, did not visualize valve vegetations but the criteria for a definitive IE according to the Duke criteria were still fulfilled [6]. We now present, to our knowledge, the first case of *S. argenteus* endocarditis with visualized valve vegetations.

## Case report

A 74-year-old male patient presented to the emergency department (ED) with a few hours’ history of fever and shivers. He stated that he had experienced an episode of dizziness leading to a minor traumatic fall without sustained injuries a couple of days prior to seeking care, but that he thenceforth felt as normal until the fever developed. Past medical history included aortic stenosis that had five years prior been surgically treated with transcatheter aortic valve implantation (TAVI) of a biological aortic valve prosthesis. He additionally had hypertension and atrial fibrillation as well as a history of prior pulmonary embolism and surgical treatment of prostate malignancy about 20 years prior without known relapse. He was presently undergoing investigations due to a mild cognitive impairment. Recent cardiac follow-up with transthoracic echocardiography (TTE) and myocardial scintigraphy had shown a left ventricular ejection fraction (LVEF) of ∼45 % and without provoked ischemia.

Upon arrival, the patient had an altered general condition but was awake and lucid. He had a body temperature of 38.5 °C, atrial fibrillation (frequency 110 beat/minute) and blood pressure within normal range. He denied a recent history of respiratory, abdominal, or urinary symptoms. External examination revealed a few minor skin ulcerations on lower extremities and a previously known basal cell carcinoma on posterior torso, all without clinical signs of infection. Neurological, heart, lung and abdominal examination were without pathological findings and no heart murmurs were audible. Initial blood test results showed a C-reactive protein (CRP) of < 4 mg/L (<5 mg/L), leukocytes of 5.5 × 10^9/L (3.5–8.8 ×10^9/L), and elevated levels of lactic acid at 4.0 mmol/L (0.5–1.6 mmol/L). Infection of an unknown origin was suspected, and blood and urine cultures were secured before administration of two grams of cefotaxime and crystalloid fluids.

The patient was admitted to the department of internal medicine and was initially in stable condition. About eight hours after admission, the patient rapidly deteriorated and was transferred to the intensive care unit due to suspicion of septic shock. His blood pressure was 80/50 mmHg, he had high frequency atrial fibrillation (120 beats per minute), respiratory rate of 30 and lactic acid levels had risen to 9.8 mmol/L (0.5–1.6 mmol/L). He was anuric with elevated transaminases, altered kidney function and coagulopathy. Norepinephrine infusion was initiated, and both crystalloid intravenous fluids and albumin were given. P-Troponin-I was elevated to > 25 000 ng/L and NT-pro-BNP to > 35 000 ng/L, chest radiography showed signs of pulmonary edema and electrocardiography (ECG) revealed newly developed anterior Q-waves, which was interpretated as a suspected type 2 myocardial infarction.

Preliminary results from blood cultures obtained at admission demonstrated growth of gram-positive cocci in clusters in all four bottles and due to a clinical suspicion of IE, cloxacillin treatment of 3 g every 6 h was initiated. The time to positive blood culture was 5 h and 57 min. *S. argenteus* was identified in all four bottles two days following admission with species determination by MALDI-TOF MS, with the MBT Compass Library, revision K (2022) (Bruker Daltonics, Bremen, Germany) combined with an in-house *S. argenteus* MSP library [7]. In addition, the presence of *S. schweitzeri* could be excluded and *S. argenteus* could be confirmed by analyzing specific peaks in mass spectra according to Schuster *et al.* and Chen *et al.* [3], [8] Antimicrobial susceptibility testing was performed with disk diffusion methodology according to the European Committee on Antimicrobial Susceptibility Testing (EUCAST) [9] using the clinical breakpoints developed for *S. aureus* [10]. The isolate was susceptible to all tested antimicrobials, including penicillin, other beta-lactams, fluoroquinolones, clindamycin, trimethoprim-sulfamethoxazole and rifampicin.

No primary source of infection was identified. Urine culture was negative for bacterial growth and unfortunately no cultures from skin ulcerations were collected. The skin ulcerations appeared as eschars without surrounding signs of inflammation or excretions and could potentially have been a possible source of infection. There were no suspicions of on-going or prior intravenous drug use and no recent history of travels or hospital admissions.

TTE the day after admission revealed LVEF of ∼30 % and signs of degeneration of the biologic aortic valve prothesis without visible valve vegetations. Transesophageal echocardiography (TEE) was delayed due to thrombocytopenia.

After four days of care the patient displayed general improvement, with stable vital signs and improved laboratory markers. Repeated blood cultures on day three after admission were negative. The patient, though, began to exhibit gradual progression of dyspnea. Bedside ultrasound and computed tomography (CT) scan of the chest showed bilateral pleural effusion and yet again troponin and NT-pro-BNP levels were rising. Treatment with loop diuretics was initiated resulting in a good clinical response. Repeated TTE was performed a week after the initial examination with improved LVEF of 40–50 % and still without visualized vegetations. TEE was further delayed due to sustained thrombocytopenia. Repeated blood cultures were once again obtained and were negative.

**Fig. 1:**
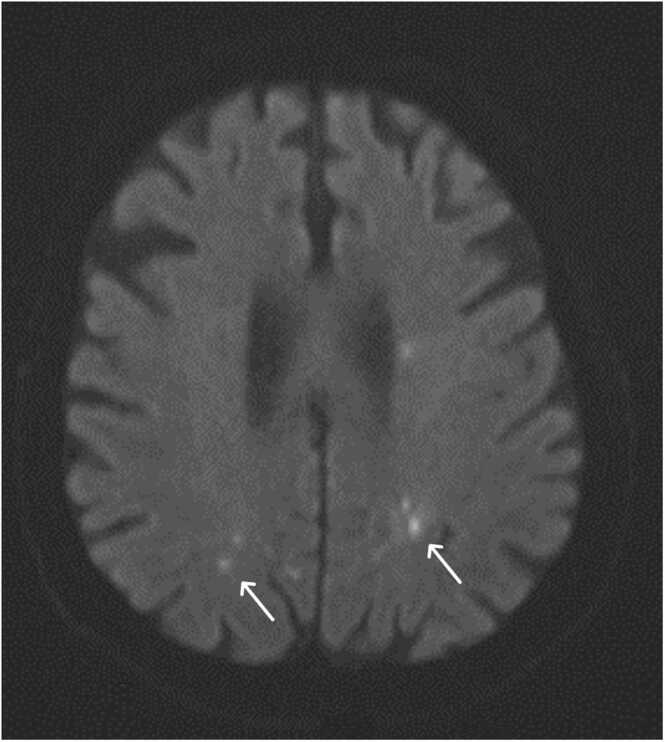
Horizontal MRI sections demonstrating selections of multiple brain lesions.

**Fig. 2:**
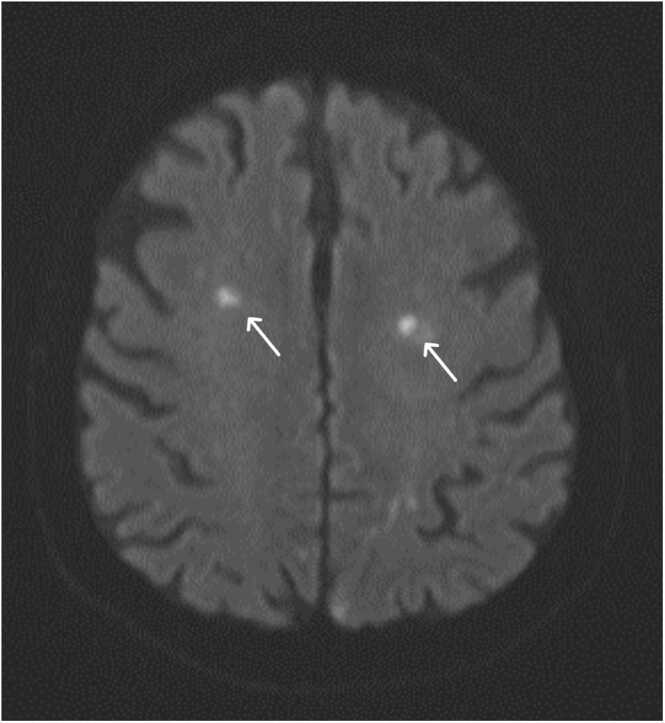
Horizontal MRI sections demonstrating selections of multiple brain lesions.

During the in-hospital stay the patient was perceived to have a fluctuating cognitive function. CT of the brain was performed showing a sub-acute ischemic lesion in right side pons. Further investigation with angiography demonstrated a 60 % stenosis of left internal carotid and Magnetic Resonance Imaging (MRI) of the brain displayed about 20 scattered small areas of around 8 mm, anatomically related to arteria cerebri media and arteria cerebri anterior (Fig. 1, Fig. 2). Due to the clinical suspicion of IE the lesions were primary suspected to represent septic emboli.

**Fig. 3:**
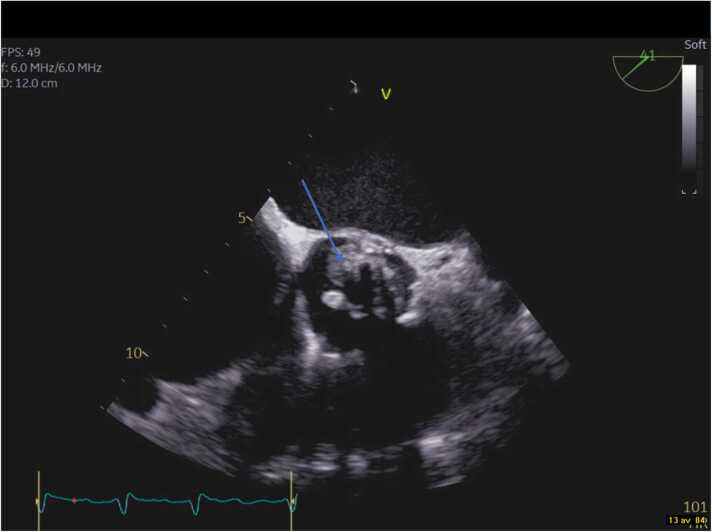
Image from transoesophageal echocardiography at mid-esophageal position. The aortic valve bioprosthesis is visualised in short-axis view, the arrow indicates the vegetation.

**Fig. 4:**
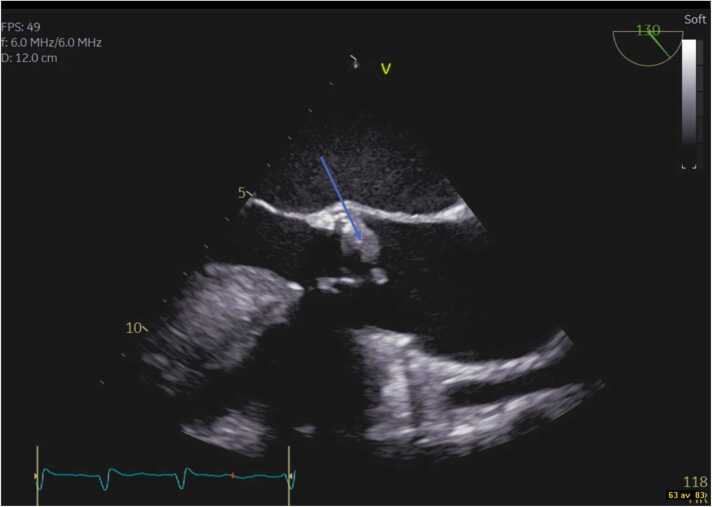
Corresponding image from long-axis view. The arrow indicates the vegetation.

About two weeks after admission the coagulopathy had improved. TEE was performed showing two large echogenic structures representing valve vegetations (measuring ∼1 × 1 and ∼1 × 0.5 cm respectively), no aortic abscess was visualized (Fig. 3, Fig. 4). The patient was following the TEE results evaluated by the endocarditis team that decided to refrain from surgical treatment. Their decision was partly based on the frail state of the patient and the multiple manifest complications which together would contribute to high surgical and anesthesiologic risks. Additionally, the valve vegetations were only visible on the TEE in contrast to the repeated TTE and at the time a TEE was possible to execute the patient was showing general improvement.

Conservative treatment continued and antibiotic regime was altered to a combination of cloxacillin and rifampicin.

The patient continued to demonstrate general clinical improvements. Laboratory markers and vital signs were improved. He was able to mobilize independently, and his cognitive functions were recovering.

Dyspnea continued to fluctuate, initially improving but then rapidly deteriorated once again. The patient was readmitted to the intensive care unit with fluctuating tachycardia, reoccurrence of fever, acute kidney injury and suspected heart failure. Norepinephrine and Levosimendan treatment and high-flow nasal cannula (HFNC) oxygen therapy was initiated and he was after stabilization transferred to the cardiac intensive care unit due to severe heart failure. Ultrasound-investigation demonstrated bilateral pleural effusions which received treatment with thoracentesis, complicated by a left sided pneumothorax. Repeated TTE demonstrated signs of valve thickening, LVEF of ∼30 % with apical hypokinesia and a left ventricular thrombus (∼25 ×10 mm). CT showed signs of right sided pneumonia and mild progression of pleural effusion. Treatment of IE, heart failure, intracardiac left ventricular thrombosis, pleural effusion, and secondary pneumonia was continued in the cardiac department where the patient slowly improved. The patient was later retransferred to the internal medicine ward and eventually discharged to his original housing situation after a total of 67 days of treatment.

The *S. argenteus* IE was treated with 6 weeks of cloxacillin and 3.5 weeks of rifampicin. Rifampicin was added to treatment around the time of the visualized valve vegetations and when 6 weeks of cloxacillin treatment had been completed the patient expressed such clinical improvements regarding the infection that both antibiotics were decided to be discontinued simultaneously. Addition of gentamicin was refrained from due to reduced kidney function.

Up to the submission of this case report the patient have continuously received follow-up by the internal medicine department of his local hospital by subspecialized cardiologists and cardiac nurses as well as a rehabilitation program with physical exercise. Repeat TTE 5 month later showed complete resolution of the thrombus, but with severely reduced LVEF of ∼20 %. At this time the patient was troubled by general fatigue but denied issues of dyspnea or chest pains. About 9 months following discharge the LVEF had improved to 30 %. He no longer experienced fatigue and was able to go for 1-kilometer-long walks without symptoms. Regular cardiac follow-up will continue in indefinite time.

## Discussion

Though *S. argenteus* is a recently defined bacterial species it has most likely been responsible for many of the infectious disease episodes previously inculpated to *S. aureus.* Modernization of laboratory methods now enables distinguishing the two bacterial species, leading to the possibilities of improved knowledge about the clinical presentation of infections caused by *S. argenteus.* Thaipadungpanit *et al.* performed typing of *S. aureus* isolates in 246 patients with invasive infection in Thailand and found that 4.1 % in fact were *S. argenteus.* They noticed a strong association with skin and soft tissue infections (8/10 cases) and found 80 % (8/10) of cases to be community-acquired [4]. A narrative review by Becker et al. from 2019 states that *S. argenteus* infections have been shown to cause skin-, soft-tissue-, bone-, joints- and blood stream-infections and that reports from Asia indicates that *S. argenteus* can cause toxin-mediated food poisoning [2]. Current knowledge of antibiotic susceptibility of *S. argenteus* is limited. A review of highly selected international material found methicillin resistance in 20 % (n = 26) of *S. argenteus* isolates [11]. The isolate of the presented case was sensitive to both isoxazolyl penicillin and penicillin.

In the presented case MALDI-TOF MS technique was applied to differentiate *S. argenteus*/*S. schweitzeri* group from *S. aureus,* followed by species-specific mass peaks correlated to an in-house MSP library to exclude the presence of *S. schweitzeri.* The applied laboratory techniques were able to successfully identify *S. argenteus* why it could be argued that this combination of techniques should be implemented as a standard. Due to the recent recognition of *S. argenteus* and the limited research, the risk of IE in *S. argenteus* bacteremia is currently unknown. Chen *et al*. performed a retrospective study comparing clinical aspects of *S. argenteus* and *S. aureus* bacteremia and demonstrated that those with *S. argenteus* not only showed a higher mortality risk but also a higher risk for thrombocytopenia [5], the latter of which also was seen in this presented case report.

The case presented herein demonstrates the risk for invasive disease and IE in *S. argenteus* bacteremia. Moreover, there were several severe complications in this case likely both related to the severity of the infection and the frailty of the patient. Our case also demonstrates that severe IE affecting prosthetic valves can sometimes be cured with conservative treatment using antibiotics alone.

The patient in the presented case had elevated levels of P-troponin-I and NT-pro-BNP and ECG showed newly developed anterior Q-waves. These cardiac findings could potentially have been explained by a coronary artery occlusion due to *inter alia* vegetation, worsening of preexisted arteriosclerotic lesion or septic embolization. In hindsight it could be speculated that the patient would have benefitted from undergoing a coronary angiography and if needed coronary revascularization. There are currently no standardized guidelines on the management of acute coronary syndrome (ACS) in IE patients, but previous presented case reports demonstrate successful results on percutaneous coronary intervention (PCI) in IE patients with ACS due to septic embolization [12], [13].

*S. argenteus* is not defined as a typical IE pathogen in the current 2023 Duke-ISCVID IE criteria [14], but with improved laboratory techniques for its identification the incidence of *S. argenteus* IE could be anticipated to increase and its inclusion in the criteria might therefore need to be reevaluated. By the time cloxacillin treatment was initiated in the presented case, the patient did not fulfill the Duke criteria for IE diagnosis, with minor criteria of fever, predisposition (prosthetic valve), and microbiological evidence falling short of major criterion (*S. argenteus* bacteremia)). If *S. argenteus* would had been classified as a major criterion for typical IE pathogen, an IE diagnosis would have been considered possible according to the criteria. Due to the eventually visualized valve vegetation and intracranial septic emboli the patient eventually met the Duke-ISCVID criteria for IE with a major imaging criterion (valve vegetation) and minor criteria of predisposition (prosthetic valve), fever, vascular phenomena (septic emboli) and microbiological evidence falling short of major criterion (*S. argenteus* bacteremia) [14].

We hope that the role of *S. argenteus* as a human pathogen will be better understood in the future, leading to improved care for patients infected by this organism.

## Author contribution

Magnus Rasmussen and Ebba Hillstedt constructed the study design, data collection and wrote original draft of the manuscript. Ebba Hillstedt, Magnus Rasmussen, Magnus Dencker, Bo Nilson and Karl Oldberg all contributed to the data collection, figures, manuscript writing and manuscript review.

## Author statement

Ebba Hillstedt treated the patient in the infectious disease ward and drafted the case report. Magnus Dencker performed echocardiography, provided expertise in this field regarding image findings and identified suitable ultrasound pictures for the report. Bo Nilson and Karl Oldberg performed microbiological tests on cultures from the patient, provided microbiological expertise and wrote parts of the case report regarding species determination and laboratory techniques. Magnus Rasmussen conceptualized the work and lead the endocarditis team in the diagnostic work-up.

All co-workers provided critical comments to the manuscript and approved the final version of the manuscript.

## CRediT authorship contribution statement

**Ebba Hillstedt:** Writing – review & editing, Writing – original draft, Project administration, Methodology, Investigation, Conceptualization. **Magnus Dencker:** Writing – review & editing, Investigation. **Bo Nilson:** Writing – review & editing, Investigation. **Karl Oldberg:** Writing – review & editing, Investigation. **Magnus Rasmussen:** Writing – original draft, Project administration, Methodology, Investigation, Conceptualization.

## Consent

The patient as well as the closest relative of the patient has given informed consent to the writing and publication of the case report.

## Ethical approval

No ethical approval has been applied for due to the format of a case report.

## Funding

No funding has been received for the research.

## Declaration of Competing Interest

The authors declare that they have no known competing financial interests or personal relationships that could have appeared to influence the work reported in this paper.

## References

[1] Tong S., Schaumburg F., Ellington M., Corander J., Pichon B., Leendertz F. (2015). Novel staphylococcal species that form part of a *Staphylococcus aureus*-related complex: the non-pigmented *Staphylococcus argenteus* sp. nov. and the non-human primate-associated *Staphylococcus schweitzeri* sp. nov. Int J Syst Evolut Microbiol.

[2] Becker K., Schaumburg F., Kearns A., Larsen A., Lindsay J., Skov R. (2019). Implications of identifying the recently defined members of the *Staphylococcus aureus* complex *S. argenteus* and *S. schweitzer*i: a position paper of members of the ESCMID Study Group for Staphylococci and Staphylococcal Diseases (ESGS). Clin Microbiol Infect: Publ Eur Soc Clin Microbiol Infect Dis.

[3] Schuster D., Rickmeyer J., Gajdiss M., Thye T., Lorenzen S., Reif M. (2017). Differentiation of *Staphylococcus argenteus* (formerly: *Staphylococcus aureus* clonal complex 75) by mass spectrometry from *S. aureus* using the first strain isolated from a wild African great ape. Int J Med Microbiol: IJMM.

[4] Thaipadungpanit J., Amornchai P., Nickerson E., Wongsuvan G., Wuthiekanun V., Limmathurotsakul D. (2015). Clinical and molecular epidemiology of *Staphylococcus argenteus* infections in Thailand. J Clin Microbiol.

[5] Chen S., Lee H., Wang X., Lee T., Liao C., Teng L. (2018). High mortality impact of *Staphylococcus argenteus* on patients with community-onset staphylococcal bacteraemia. Int J Antimicrob Agents.

[6] Hirai J., Suzuki H., Sakanashi D., Kuge Y., Kishino T., Asai N. (2022). The first case report of community-acquired infective endocarditis due to sequence type 1223 *Staphylococcus argenteus* complicated with convexity subarachnoid hemorrhage. Infect Drug Resist.

[7] Tjörnstrand U., Seid H., Petersson A.-C., Nilson B. Improved species identification of *Staphylococcus argenteus* with MALDI-TOF MS using an extended MSP library. Manuscript.

[8] Chen S.Y., Lee H., Teng S.H., Wang X.M., Lee T.F., Huang Y.C. (2018). Accurate differentiation of novel *Staphylococcus argenteus* from *Staphylococcus aureus* using MALDI-TOF MS. Future Microbiol.

[9] Matuschek E., Brown D.F., Kahlmeter G. (2014). Development of the EUCAST disk diffusion antimicrobial susceptibility testing method and its implementation in routine microbiology laboratories. Clin Microbiol Infect.

[10] The European Committee on Antimicrobial Susceptibility Testing. Breakpoint tables for interpretation of MICs and zone diameters. Version 13.1, 2023. 〈http://www.eucast.org〉. [Internet].

[11] Goswami C., Fox S., Holden M., Leanord A., Evans T.J. (2021). Genomic analysis of global *Staphylococcus argenteus* strains reveals distinct lineages with differing virulence and antibiotic resistance gene content. Front Microbiol.

[12] Sugi K., Nakano S., Fukasawa Y., Maruyama R., Tanno J., Senbonmatsu T. (2015). Percutaneous coronary intervention for septic emboli in the left main trunk as a complication of infective endocarditis. Heart, lung Circ.

[13] Mazzoni C., Scheggi V., Marchionni N., Stefano P. (2021). ST-segment elevation myocardial infarction due to septic coronary embolism: a case report. Eur Heart J Case Rep.

[14] Fowler V., Durack D., Selton-Suty C., Athan E., Bayer A., Chamis A. (2023). The 2023 duke-international society for cardiovascular infectious diseases criteria for infective endocarditis: updating the modified duke criteria. Clin Infect Dis: Publ Infect Dis Soc Am.

